# Development of Polythiourethane/ZnO-Based Anti-Fouling Materials and Evaluation of the Adhesion of *Staphylococcus aureus* and *Candida glabrata* Using Single-Cell Force Spectroscopy

**DOI:** 10.3390/nano11020271

**Published:** 2021-01-21

**Authors:** Sophie Klemm, Martina Baum, Haoyi Qiu, Zibin Nan, Mafalda Cavalheiro, Miguel Cacho Teixeira, Claire Tendero, Anna Gapeeva, Rainer Adelung, Etienne Dague, Mickaël Castelain, Cécile Formosa-Dague

**Affiliations:** 1 Functional Nanomaterials, Institute for Materials Science, Kiel University, 24143 Kiel, Germany; sophie.klemm@tu-berlin.de (S.K.); haq@tf.uni-kiel.de (H.Q.); ang@tf.uni-kiel.de (A.G.); ra@tf.uni-kiel.de (R.A.); 2 LAAS-CNRS, Université de Toulouse, CNRS, 31400 Toulouse, France; edague@laas.fr; 3 TBI, Université de Toulouse, INSA, INRAE, CNRS, 31400 Toulouse, France; nan@insa-toulouse.fr (Z.N.); castelai@insa-toulouse.fr (M.C.); 4 Institute for Bioengineering and Biosciences (iBB), Instituto Superior Técnico, University of Lisbon, 1049-001 Lisbon, Portugal; mafalda.cavalheiro@tecnico.ulisboa.pt (M.C.); mnpct@tecnico.ulisboa.pt (M.C.T.); 5 CIRIMAT, Université de Toulouse, CNRS, INPT, UPS, 31400 Toulouse, France; claire.tendero@inp-toulouse.fr; 6 Fédération de Recherche Fermat, CNRS, 31000 Toulouse, France

**Keywords:** polythiourethane, tetrapodal shaped ZnO, PTU/ZnO composite, microbial adhesion, initial attachment, *S. aureus*, *C. glabrata*, single cell force spectroscopy, anti-fouling

## Abstract

The attachment of bacteria and other microbes to natural and artificial surfaces leads to the development of biofilms, which can further cause nosocomial infections. Thus, an important field of research is the development of new materials capable of preventing the initial adhesion of pathogenic microorganisms. In this work, novel polymer/particle composite materials, based on a polythiourethane (PTU) matrix and either spherical (s-ZnO) or tetrapodal (t-ZnO) shaped ZnO fillers, were developed and characterized with respect to their mechanical, chemical and surface properties. To then evaluate their potential as anti-fouling surfaces, the adhesion of two different pathogenic microorganism species, *Staphylococcus aureus* and *Candida glabrata,* was studied using atomic force microscopy (AFM). Our results show that the adhesion of both *S. aureus* and *C. glabrata* to PTU and PTU/ZnO is decreased compared to a model surface polydimethylsiloxane (PDMS). It was furthermore found that the amount of both s-ZnO and t-ZnO filler had a direct influence on the adhesion of *S. aureus*, as increasing amounts of ZnO particles resulted in reduced adhesion of the cells. For both microorganisms, material composites with 5 wt.% of t-ZnO particles showed the greatest potential for anti-fouling with significantly decreased adhesion of cells. Altogether, both pathogens exhibit a reduced capacity to adhere to the newly developed nanomaterials used in this study, thus showing their potential for bio-medical applications.

## 1. Introduction

Hospital-acquired microbial infections, also called nosocomial infections, are nowadays a leading cause of death on all continents [1]. Such infections are often caused by pathogenic bacteria and yeast, which are able to form biofilms on medical devices such as catheters, pacemakers or prosthetic joints. Biofilms are structured microbial communities that show increased resistance to antibiotics [2,3]. Such medical device-associated infections represent a heavy burden for societies, as they are responsible for increasing the length of hospital stay for patients and result in high mortality rates [4]. Biofilm formation on surfaces usually involves three steps. The first step consists of the reversible adhesion of the microbial cells to surfaces involving physico-chemical forces. It is then followed by a transition from reversible to irreversible microbial adhesion where physico-chemical bonds are strengthened [5] and chemical forces, such as hydrophobic or hydrophilic forces [6], can be involved. Finally, after these adhesion steps, cells undergo important changes (cell wall deformation, production of exopolysaccharides, gene expression etc.) that lead to the formation of a mature biofilm [5]. 

So far, diverse strategies have been explored by scientists to combat the formation of biofilms on abiotic surfaces [7]. Such strategies include the use of anti-biofilm treatment agents, such as specific enzymes targeting the exopolysaccharide (EPS) matrix of mature biofilms [8], anti-quorum sensing molecules that interfere with biofilm development [9], and anti-oxidants that inhibit biofilm formation in many bacterial species [10]. Other studies have focused on developing anti-biofilm strategies based on the inhibition of cell–cell interactions, which also play a crucial role in biofilm formation [11,12].Further, biofilm formation can be prevented by focusing on the surface properties of the material itself. From this point of view, anti-adhesive surfaces that directly inhibit the first steps of biofilm formation, i.e., cell adhesion, have been developed over the years. Some of these strategies include surface coatings with silver nanoparticles [13] or with polymeric hydrophilic materials [14,15]. Hydrophilic coatings prevent microbial adhesion thanks to a tightly correlated water layer that builds up on the material surface and forms an energetic barrier against microbial cells. In general, Geoghegan et al. showed stronger adhesion to hydrophobic materials for several bacterial strains (*Aquabacterium commune, Escherichia coli, Rhodococcus* spp., *Pseudomonas* sp., *Acidovorax* sp.) [16]. However, several studies have shown that hydrophobic or superhydrophobic properties of textured surfaces were able to reduce bacterial adhesion [17,18], for example in the cases of *S. aureus*, *E. coli*, or *Staphylococus epidermidis*. Another well investigated mechanism involves steric repulsion. Polyethylene glycol is considered a gold standard polymer able to reduce adhesion via hydration forces and steric hindrance [19,20]. However, its oxidation in biological environments needs to be considered as it can be harmful to the human body [21]. As the surface roughness of a material also interferes with the adhesion, by changing the availability of the contact surface area [15,22], surface patterning is another strategy to combat initial cell attachment [14,20,21,22]. Several studies have reported that the patterning of titanium (Ti) surfaces in the nanometer range influences the adhesion of *S. aureus* [23,24,25]. However, while Ercan et al. observed reduced adhesion with increasing roughness (from 60 to 80 nm) [23], Wang et al. showed that for TiO_2_, the increase in roughness from 70 nm to 120 nm led to an increase in cell adhesion by three-fold [26]. These contradicting results show that the interpretation of the effects of physical parameters on the adhesion process is difficult and should be accompanied by chemical analysis to provide a full understanding. Furthermore, these examples of strategies to produce anti-fouling surfaces show that, so far, no ideal strategy has been developed to combat biofilm formation of multiple species; the research towards new materials to overcome this challenge has to be actively pursued.

As stated previously, the physical and chemical properties of the materials surface are of great importance. Therefore, the anti-fouling materials we developed in this work are based on a polymer/ceramic system. We combined polythiourethane (PTU) as a matrix polymer with zinc oxide (ZnO) particles of varying morphology and amount. The variation of morphology and amount of ZnO particles allows us to alter the topographical and physico-chemical properties of PTU. PTU is a two-component, full-solid, non-toxic, transparent thermoset polymer system, which is also resistant to UV-irradiation [27] and biocorrosion [28,29,30]. PTU forms very smooth surfaces (R_a_: 0.037 µm) and does not swell in an aqueous environment [31]. Furthermore, PTU is easy to fabricate by a simple, solvent-free polyaddition reaction [28], and was successfully tested as an anti-fouling coating for ship hulls [28,31,32,33]. ZnO particles and ZnO thin films are widely used for various industrial applications, e.g., in optoelectronics, piezo electronic devices or anti-viral products [34,35]. Furthermore, the anti-microbial activity of ZnO nanoparticles is widely known and has been strongly investigated [34,36]. In this study we used tetrapodal ZnO microparticles next to spherical particles. T-ZnO has the advantage of bearing all ZnO’s intrinsic properties, e.g., high Young’s modulus and strength as well as biocompatibility [34], but with the added benefits of an even further reduced toxicity [37], improved force transduction [34,38] and anti-fouling properties [28]. The addition of t-ZnO particles allows for the modification of the composite material in terms of strength, elasticity and surface roughness which allows a broader spectrum of potential applications in the human body.

In the present study, we characterized the material properties of the developed PTU/ZnO composites (including PTU without addition of ZnO particles) in terms of surface roughness, mechanical properties, surface charge and hydrophobicity; properties that have been demonstrated to influence microorganism adhesion [39]. Their anti-adhesive properties towards two medically relevant microbial species, *S. aureus* and *C. glabrata*, were then evaluated using atomic force microscopy (AFM) [40]. To achieve this, *S. aureus*, the Gram-positive bacterial species most frequently found in health-care associated infections [41], and *C. glabrata*, a yeast species with dramatic implications in immune-suppressed patients [42], were used in single-cell force spectroscopy [43] experiments to probe their direct interactions with the different hydrophilic PTU/ZnO composites. Polydimethylsiloxane (PDMS) was used in this study as a hydrophobic model surface as it is widely used for medical devices [44,45,46]. These experiments allowed the comparison of the bio-adhesive properties of each material depending on the type of microorganism, but have also enlightened the molecular and biophysical mechanisms involved in such interactions. Altogether, this work provides a step forward towards tailoring efficient anti-adhesive materials that can be used in future biomedical applications.

## 2. Materials and Methods

### 2.1. Polymer Composites Preparation

The investigated PTU/ZnO composites are based on a two-component PTU thermoset (Fluid-& Prozesstechnik GmbH, Waltershausen, Germany), which consists of pentaerythritol tetrakis (3-mercaptopropionate) (PETMP) and hexamethylene diisocyanate (HDI). Respectively, 1 and 5 wt.% ZnO particles were dispersed within PETMP component by the MiniMaster Laboratory Disperser (Netzsch-Feinmahltechnik GmbH, Selb, Germany) with a dispersion speed of 6000 rpm for 15 min. Subsequently, the second component, HDI, was added and stirred by hand for approx. 1 min. The ratio of PETMP to HDI was kept at 1 to 1.4 in weight for all PTU/ZnO composites [31]. Hölken et al. showed that the ratio of 1:1.4 PETMP:HDI results in the highest ultimate tensile strength for this polymer system [28]. After degassing the polymer blend by using a desiccator, the final mixture was cast into silicone molds (9 × 30 × 0.9 mm) and cured at 85 °C for 24 h under atmospheric pressure. Detailed information on the polymerization reaction of PTU can be found in Hölken et al. 2016 [28]. To investigate the influence of the amount of ZnO particles and their geometry on bio-adhesive properties of the polymer composites, 1 and 5 wt.% of tetrapodal (t-ZnO) and spherical (s-ZnO) ZnO particles were added. T-ZnO was produced by flame transport synthesis at Kiel University, Kiel, Germany [47,48]. The four arms of the t-ZnO form angles of 109.5° to each other, have a length of ca. 15–20 µm and a diameter of around 3–5 µm. S-ZnO with a grain size of 1–10 µm was purchased from Sigma-Aldrich Chemie GmbH, Munich, Germany. SEM images of both types of particles can be seen in [28]; SEM images of the different PTU/ZnO composites produced in this study are presented in Figure S1 (Supplementary Materials). 

As a hydrophobic reference material, a two-component silicone rubber (ADDV-25, R&G Faserverbundwerkstoffe GmbH, Waldenbuch, Germany) was used and the samples were cast in the same dimensions and under the same conditions as for the PTU/ZnO composites.

### 2.2. AFM Surface Imaging

The nanoscale surface roughness of the PTU/ZnO composites and PDMS was performed using a Nanowizard III AFM (Bruker, Billerica, MA, USA) with MLCT AUWH probes (Bruker, Billerica, MA, USA, nominal spring constant of 0.6 N/m). The spring constant of the cantilevers was determined prior to imaging using the thermal noise method and ranged from 0.447 to 0.540 N/m [49]. Three images (20 × 20 µm^2^) of each PTU/ZnO composite and the PDMS specimens were recorded in contact mode in air with a constant applied force of 2 nN and a line rate of 3.0 Hz. Figure S2 (Supplementary Materials) show examples of AFM height images recorded for each type of materials (PDMS and PTU/ZnO composites). For roughness analysis, the arithmetic roughness (*R_a_*) was calculated using JPK Data Processing Software on flattened (order 3) high-resolution (512 × 512 lines) height images, following Equation (1).

(1) $$Ra=\frac{1}{L}\int_{0}^{L}\left|z\left(x\right)\right|dx$$

where *L* is the evaluation length and *z*(*x*) the profile height function. 

### 2.3. Confocal Laser Scanning Microscopy (CLSM) Imaging

To obtain the mean arithmetic roughness (*R_a_*), the 3D Laser Scanning Confocal Microscope VK-X (Keyence Corporation, Osaka, Japan) with a red semiconductor laser source (wavelength of 658 nm) was used. For roughness determination, a magnification 100× was used. For obtaining the roughness, a total of ten-line profile measurements were conducted in x-and y-directions for PTU/ZnO composites and PDMS. The line profile was conducted on an image with the size of 1 × 1.5 mm^2^.

### 2.4. Microorganism Strains and Culture Conditions

The bacterial strain *S. aureus* ATCC 19123 and the yeast strain *C. glabrata* KChr606 were used in this study. *S. aureus* cells were cultured in Mueller–Hinton (MH) broth (Difco, Kansas, USA) at 37 °C under agitation (150 rpm) overnight. For the AFM experiments, bacterial cells were harvested by centrifugation (1000× *g*, 3 min) and washed two times in PBS 1X (phosphate-buffered saline, pH 7.4). *C. glabrata* cells were cultured in yeast extract peptone dextrose (YPD) broth (Difco, Kansas, USA) at 30 °C under agitation (250 rpm) overnight. For AFM experiments, the yeast cells were harvested by centrifugation (5000 rpm, 5 min) and washed two times in acetate buffer (1.48 g/L sodium acetate, 111 mg/L CaCl_2_ and 198 mg/L MgCl_2_, 5.2 pH).

### 2.5. Single-Cell Force Spectroscopy Experiments

These experiments were performed using a Nanowizard III AFM (Bruker, Billerica, MA, USA) equipped with an inverted light microscope (Zeiss, Jena, Germany). NP-O10 probes (Bruker, Billerica, MA, USA) with a nominal spring constant of 0.06 N/m for experiments with *S. aureus* and of 0.35 N/m for experiments with *C. glabrata* were used to measure the interactions between single-cells and the different material surfaces. For this, given their respective sizes *S. aureus* (~1 µm of diameter), cells were first immobilized on colloidal probes [43], while *C. glabrata* cells (~4–5 µm in diameter) were immobilized directly on the tipless cantilevers. All cantilevers were cleaned using oxygen plasma before use. For all conditions, force maps of 10 × 10 pixels were recorded on 1 × 1 µm² areas on the surfaces. In the case of *S. aureus*, colloidal probes were obtained by attaching a single silica microsphere (5 μm diameter, Bangs Laboratories, Fishers, Indiana, IN, USA) with a thin layer of UV-curable glue (NOA 63, Norland Edmund Optics, Barrington, NY, USA) on the cantilevers using the AFM. Afterwards these were immersed for 1 h in tris buffer saline (TBS, pH 8.5) containing 4 mg/mL of dopamine hydrochloride (Sigma-Aldrich Chemie GmbH, Munich, Germany), rinsed in PBS 1X, and used directly for cell probe preparation. The spring constant of the colloidal probes, ranging from 0.0554 to 0.0932 N/m was determined prior to cell immobilization using the thermal noise method [49]. Post preparation, the colloidal probe was brought into contact with an isolated bacterium and subsequently retracted with the attached bacterial cell. The attachment of the cell on the silica microsphere was confirmed using optical microscopy and by performing a force distance curve on the Petri dish. The maximum force applied equaled 250 pN and a constant approach and retraction velocity of 1 µm/s was applied. For each condition, experiments were repeated for seven cells originating from at least three different cultures, and 400 force curves were recorded for each cell. 

In the case of *C. glabrata* cells, the cantilevers were incubated in a solution of Concanavalin A (ConA, Sigma-Aldrich, 0.1 mg/mL) overnight and rinsed in acetate buffer. The spring constant of the ConA functionalized cantilevers, ranging from 0.213 to 0.361 N/m, was determined prior to cell attachment using the thermal noise method [49]. The ConA cantilever was then brought into contact with an isolated yeast cell and retracted with the attached yeast cell. In this case also the proper attachment of the cell on the cantilever was confirmed using an inverted light microscope (Zeiss, Jena, Germany). In contrast to experiments with *S. aureus,* tipless cantilever can be used without a sphere because of the larger size of the *C. glabrata* cells. The maximum force applied equaled 1000 pN and a constant approach and retraction velocity of 5 µm/s was applied. For each condition, experiments were repeated for seven cells, originating from at least three different cultures, and 400 force curves were recorded for each cell.

For both types of cell, probes were finally used to measure cell–surface interaction forces in PBS 1X for *S. aureus*, and in acetate buffer for *C. glabrata*, both at room temperature. Data were analyzed using the JPK Data Processing software (Bruker, Billerica, MA, USA). Adhesion force and rupture distance histograms were obtained by calculating the maximum adhesion force and the rupture distance of the last peak for each obtained curve. The mean values and standard deviations were calculated and the significance was tested by performing a two sample t-test by using OriginLab (OriginLab Corporation, Northampton, MA, USA). The force distance curves were finally analyzed by using the JPK Data Processing software (JPK Instruments, Berlin, Germany). The adhesion force and the rupture distance were obtained and summarized in histograms with a bin interval of 50 pN and 20 nm for *S. aureus* and 100 pN and 50 nm for *C. glabrata*, respectively. The mean values and standard deviations were calculated using OriginLab (OriginLab Corporation, Northampton, MA, USA).

### 2.6. Tensile Testing

In the underlying study, a Zwick 1445 universal testing machine (Zwick GmbH& Co.KG, Ulm, Germany) was used to perform the tensile tests (ISO 527) on the PTU/ZnO composites with an initial load of 5 N and an applied constant strain rate of 1 mm/min. For testing PDMS, the initial load was set to 0.1 N and the strain rate to 20 mm/min. The samples were prepared by the mixture casting into a dog-bone shaped silicone mold. The effective dimensions of the samples were 20 mm in length, 5 mm in width and 1 mm in thickness. The Young’s Modulus was calculated from the stress–strain relationship using Hooks Law below (2), as described in [31]. The mean values and standard deviations of ten samples for every PTU/ZnO composite and PDMS were calculated using OriginLab (OriginLab Corporation, Northampton, MA, USA).

(2) $$E=\;\frac{\sigma }{\varepsilon }$$

where *E* is the elastic modulus, *σ* is the stress and *ε* the strain.

### 2.7. Scanning Electron Microscopy (SEM) Imaging and Energy Dispersive X-ray Spectroscopy (EDX)

To evaluate the presence of chemical elements like Zn, a SEM (Zeiss ULTRA PLUS microscope with GEMINI column, Carl Zeiss AG, Oberkochen, Germany) equipped with an InLens detector and an EDX-detector (Carl Zeiss AG, Oberkochen, Germany) was used to investigate the PTU/ZnO composites. The acceleration voltage was set to 10 keV and the magnification to 300×. The topside of the casted samples was investigated. The polymer material was sputter coated with gold by a BAL-TEC SCD 050 Sputter Coater (Bal-Tec AG, Pfäffikon, Switzerland) for 80 s with a current of 30 mA. The amount of C and Zn detected by EDX on one sample was used to calculate the Zn/C ratio presented in Table S1 in the Supplementary Materials section.

### 2.8. Photometric Determination of Potential Zn-Ion (Zn^2+^) Release

Potential Zn^2+^ release from the PTU/ZnO composites was investigated by a photometric method, which is based on the reaction of zincon (2-carboxy-2′-hydroxy-5′-sulfoformazylbenzene). This chemical complex binds Zn^2+^ and forms a blue complex at pH 8.5–9.5. Prior to the Zn^2+^ concentration measurements, PTU/ZnO composites were washed with 2-propanol to remove potential superficial contaminations. To simulate a short-time exposure of those materials to an aqueous environment, the composite materials were immersed in deionized water for one day; for long-time exposure for 27 days. To investigate the potential Zn^2+^ release from PTU/ZnO composites, the topside and backside of the cast specimens with 1 wt.% and 5 wt.%, s- and t-ZnO were tested individually.

The 0.1 wt.% zincon solution contained 13 mg zincon in 0.2 mL 1 M NaOH solution and 10 mL deionized water. 0.1 mL of the aqueous test solution (incubated with the PTU/ZnO composites) was mixed with 0.3 mL zincon solution. The pH was adjusted by borax buffer (Na_2_B_4_O_7_x10H_2_O (pH 9)). Deionized water was added to obtain a total volume of 5 mL. The intensity of the blue color was measured photometrically, by an Epoch 2 Microplate Spectrophometer (BioTek Instruments Inc., Winooski, USA) at a wavelength of 620 nm. A calibration curve was constructed to assess the concentration of Zn^2+^ in tested samples. The amount of Zn^2+^ detected is presented in Table S2 in the Supplementary Materials section.

### 2.9. Wetting Properties

The water contact angle measurements were determined with the Drop Shape Analyser 25B (A. KRÜSS Optronic GmbH, Hamburg, Germany) by the sessile drop method with a drop volume of 8 μL. Three surfaces were investigated for each PTU/ZnO composition and for PDMS and three measurements were conducted on each surface (nine measurements per material variation). The contact angle gives information about the wettability of a surface. If the contact angle exceeds 90° the surface is considered hydrophobic otherwise the surface is considered hydrophilic.

### 2.10. Surface Charge Evaluation

The Zeta potential ζ was measured on the surface of flat samples with a ZetaCAD^®^ (CAD Instruments, Les Essarts le roi, France) setup. The various samples were immersed for one hour in KNO_3_ 10^−3^ M before being inserted in the flow cell. A pressure ramp (from 20 to 235 mBar with a step of 20 mBar) was applied to the flow cell. At each pressure step the electrolyte flows in two directions (from tank 1 to 2 and inversely). For each step and each flow direction, the resulting streaming potential was measured. The streaming potential is linked to the zeta potential ζ through the Helmholtz–Smoluchowski equation [50]. Electrolyte pH, temperature and conductivity were measured for each experiment. 

The values of the zeta potential are robust as far as both repeatability and reproducibility are concerned: each sample was tested three times and each kind of surface was evaluated with three couples of samples. Therefore, the streaming potential values given for each material variation result from nine measurements. The global deviation includes both correlation and experimental deviation.

## 3. Results and Discussion

### 3.1. PTU/ZnO Composites Have Anti-Adhesive Surface Properties

Given the importance it may have for cell adhesion, the first property of the PTU/ZnO composites that we chose to explore in this study was surface roughness. For this, we used two established techniques to characterize the surface materials, confocal laser-scanning microscopy (CLSM) and atomic force microscopy (AFM) [51], and we measured the average roughness R_a_ of the PTU/ZnO composites and the PDMS specimen. The data obtained are presented in Table 1.

The roughness analysis using CLSM was performed on images of 1 × 1.5 mm^2^, while for AFM it was performed on images of 20 × 20 µm^2^. Thus, the area scanned using CLSM was 3750 times larger than the area scanned using AFM, explaining the differences observed in the values between the two techniques. The first interesting point to note, is that while CLSM measurements gave a similar value for the roughness of PTU and PDMS, measurements at the micro-scale with AFM show an important difference between PDMS and PTU, with a roughness value of 13.6 ± 4.6 nm for PDMS and of 0.401 ± 0.075 nm for PTU. This difference may, indeed, be due to the size of the areas scanned in each case, but also to the different detection methods involved in the two techniques (CLSM, reflection of visible light; AFM, physical deflection of a cantilever). However, both techniques show the same tendency: while the addition of 1 wt.% of spherical ZnO particles tends to smoothen the surface, although the difference with PTU in both cases is not significant, increasing the amount of particles from 1 to 5 wt.% increases the roughness of the material. In the situation where tetrapodal particles are added to PTU, the roughness is slightly increased when 1 wt.% of particles were added compared to PTU; this increase is more pronounced with the addition of 5 wt.% of particles. It is important to note that in all cases, no Zn^2+^ was found at the surface of the materials, neither by EDX analysis nor by Zn^2+^ release measurements (Supplementary Tables S1 and S2), which implies that the roughness differences observed are only influenced by the filling material, and not by the physical presence of ZnO particles at the surface. The literature on the influence of roughness on cell adhesion is conflicting. While some studies suggest that an *R_a_* smaller than 200 nm does not affect adhesion [52], different authors showed differences in the adhesion of cells on nanorough surfaces (30 nm to 120 nm) [20,23]. However, as far as we know, the roughness in the sub-nanometer range has not been extensively investigated in the context of microbial adhesion. Thus, it is difficult to state at this stage whether the small roughness differences measured between the PTU variations will influence cell adhesion. 

Another component considered in this study is the material stiffness. In material science, material stiffness is a basic parameter, most relevant in evaluating fundamental phenomena like adhesion, friction or crack propagation [51,53]. However, within the interdisciplinary field of bio-adhesion between abiotic surfaces and microorganisms, this issue has not yet been widely addressed, although it has been shown that the stiffness of a material can influence the adhesion of bacteria onto it [39]. In the case of the PTU/ZnO composites produced in this study, the stiffness of the material variations was measured using tensile tests; the results are presented in Table 2. They show that while the stiffness of PTU is five times higher than that of PDMS, there are no significant differences observed between the PTU variations, which is in accordance with previous measurements performed on other batches of PTU/ZnO materials produced in our team [28]. Only PTU with 5 wt.% of t-ZnO shows a significant difference to the other PTU variations; this might be due to the geometric arrangement of the tetrapods in the material. If we put these results in light of the study by Song and Ren, then the adhesion to the stiffer material, namely PTU and PTU/ZnO variations, should be less favored by the microorganisms tested in this study. 

The hydrophobicity of a material is also a crucial factor to consider when evaluating the adhesion of microorganisms to artificial surfaces [54,55,56]. For instance, it has been already shown that microorganisms such as *S. aureus* or *C. glabrata*, used in this study, adhere more to hydrophobic surfaces, as do many other microbes [17,57,58,59,60,61]. Thus, we characterized the wetting properties of the PTU/ZnO variation compared to PDMS; the results are presented in Table 3. Figure S3 (Supplementary Materials) shows examples of the corresponding micrographs from which the measurements were performed. While PDMS is hydrophobic with a Water Contact Angle (WCA) of 114.9 ± 1.4°, PTU and PTU/ZnO variations are hydrophilic (WCA comprised between 78.6–73.3°). No significant differences can be observed among the PTU/ZnO variations. As well as for the increased stiffness, the hydrophilic properties of PTU/ZnO composites should also lead to the reduced adhesion of microbial cells. 

Finally, the last parameter we measured to characterize the PTU/ZnO composites was their surface charge. Indeed, during the first phase of cell adhesion to an abiotic surface, electrostatic interactions are at play; alongside to van der Waals and hydrophobic forces, they mediate non-specific cell adhesion [61]. For this reason, notably, the surface charge is one of the most studied cell wall properties of bacteria cells [15]. The net surface charge of microbial cells in physiological medium is negative [62]. To evaluate the strength of electrostatic interactions between *S. aureus* respectively *C. glabrata* and the PTU/ZnO compositesthe **ζ**-potential of PDMS, PTU and PTU with 5 wt.% of spherical and tetrapodal ZnO particles was measured: the results are presented in Table 4. The results show that all the surfaces are negatively charged; no significant differences can be observed between PTU and PTU functionalized by 5 wt.% ZnO, whether of spherical or tetrapodal filler shape. An important point to note, is that the PDMS surface is six times less charged than PTU: thus, on this surface there will be less repulsion with cells than for PTU and PTU variations, which may also have an important influence on cell adhesion. Indeed, as the cell wall of microbial cells is negatively charged, the repulsion forces will increase with increasing the negative charge of the material surface.

All these experiments have thus allowed us to characterize the properties of the PTU/ZnO composites investigated in this study, and to compare them with the ones of PDMS. Our analyses show that PTU/ZnO composites have a decreased roughness compared to PDMS, an increased stiffness, they have a lower WCA and are hydrophilic, and finally, they present a more negatively charged surface. Within the PTU variations, the only significant difference was found concerning the stiffness, where PTU with 5 wt.% of tetrapodal ZnO particles had an increased stiffness compared to the other PTU variations. Based on these findings, the potential of these materials to reduce microbial adhesion, atomic force microscopy was used to probe the interactions between microbial cells and the different material surfaces at the molecular scale.

### 3.2. Adhesion of S. aureus to PTU/ZnO Composites Is Significantly Decreased Compared to PDMS

To evaluate whether the PTU/ZnO polymer variations are efficient anti-fouling materials, single-cell force spectroscopy experiments were conducted first using *S. aureus* cells. In this type of experiment, a single living bacterial cell is attached to a colloidal cantilever and directly used, in force spectroscopy mode, to probe the interactions with the surface. This gives access to matrices of force–distance curves recorded over a micrometer-sized (1 × 1 µm²) area of the surface, from which different information, such as the maximum adhesion peak and the rupture distance, can be extracted. The maximum adhesion peak quantifies the maximum force of the interaction, and the rupture distance gives information on the length of the unfolded molecules at the surface of the cell, and thus, on the nature of the interaction probed. Moreover, the percentage of adhesion is determined by the number of force curves presenting adhesive events in each case. 

**Figure 1:**
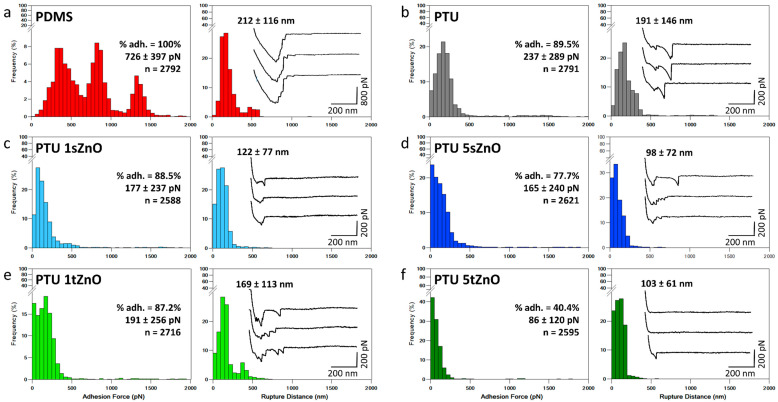
Single-cell force spectroscopy to probe the interactions between *S. aureus* and material surfaces. Adhesion force histogram and corresponding rupture distance histogram obtained for seven cells interacting with (**a**) PDMS, (**b**) PTU, (**c**) PTU containing 1 wt.% of s-ZnO, (**d**) PTU containing 5 wt.% of s-ZnO, (**e**) PTU containing 1 wt.% of t-ZnO and (**f**) PTU containing 5 wt.% of s-ZnO. Insets in rupture distance histograms show representative force curves obtained in each case. % adh. is the % of force curves presenting adhesive events. Data were recorded using a set-point of 0.25 nN. 3.3 PTU/ZnO composites are also efficient anti-adhesive surfaces against yeast cells.

The histograms of the results obtained are presented in Figure 1. For each type of polymer composite and PDMS, the interactions with seven different cells, coming from at least three independent cultures are presented. The results obtained show that PDMS presents the highest average adhesion force of 726 ± 397 pN, as well as the highest average rupture distance of 212 ± 116 nm among the material variations, containing PTU/ZnO composites and PDMS (Figure 1a). Compared to PDMS, PTU shows a significantly lower number of adhesive events (89.5% instead of 100% for PDMS), a lower average adhesion force with 237 ± 289 pN and a corresponding average rupture distance of 191 ± 146 nm (Figure 1b). With the addition of ZnO particles into the PTU, the number of force curves presenting adhesive events and adhesion forces decrease, as well as the corresponding rupture distances. The minimum average adhesion force recorded, of 86 ± 120 pN, is reached for PTU with 5 wt.% t-ZnO (only 40.4% of adhesive curves, Figure 1f). The average rupture distance for PTU with 5 wt.% t-ZnO is 103 ± 61 nm, which is comparable to PTU containing 5 wt.% s-ZnO. These results are also summarized in Table 5. Statistical analysis (two-sample t-test) showed that the differences recorded in the adhesion forces are significantly different (*p*-value < 0.001), with only one exception made by PTU with a filler content of 1 wt.% of t-ZnO, for which the adhesion force is not significantly different from PTU and the other PTU/ZnO composite variations. From these results it thus seems that PTU variations display anti-adhesive properties as the number of adhesive events and the adhesion force values are significantly decreased compared to PDMS. While this was known for classic polyurethane materials coatings [63], it is the first time this has been shown for PTU. Moreover, the increasing amount of both types of ZnO particles present in the PTU further decreases both the frequency of adhesion of *S. aureus*; while in the case of s-ZnO particles, the adhesion force does not significantly decrease with the increasing amount of particles, in the case of t-ZnO, the adhesion force is reduced by a factor of 2.2. Therefore, it seems the tetrapodal shape of ZnO particles is more efficient at decreasing the adhesion of cells. The significant decrease in the adhesion forces indicate the reduced bond-strengthening on PTU/ZnO composites; thus, irreversible adhesion is weaker. This proves the suitability of PTU/ZnO composites as anti-adhesive surfaces in the case of *S. aureus*.

The rupture distances obtained for PTU/ZnO composites are rather long and indicate that the interactions between the cells and the surfaces do not rely only on physico-chemical forces, but also on specific interactions involving specific cell-surface polymers. Indeed, when looking at the retract force curves obtained, multiple peaks can be observed after the contact point, thus proving that molecules at the surface of cells interact with the material and are unfolded upon retraction. Such molecules could be surface proteins or strands of peptidoglycan present on the surface of Gram-positive bacteria such as *S. aureus*, interacting with the polymer surface and thus strengthening the initial physico-chemical bonds between cells and surfaces, to transition to an irreversible adhesion [5]. 

The reasons explaining the decrease in adhesion force can be directly correlated to the characteristics of the surfaces described earlier. The first reason, is that PTU variations, compared to PDMS, are hydrophilic (for details see Table 3). Indeed, several studies have shown that an increased hydrophobicity of the material was responsible for a high level of bacterial initial binding to the surface [56,64]. A study conducted using *S. epidermidis* even showed that an increase in the degree of hydrophobicity of materials was linearly correlated with the number of adherent bacterial cells on the surface [57]. The propensity of *S. aureus* cells to adhere more to a hydrophobic surface such as PDMS compared to the hydrophilic PTU/ZnO variations, may be due to the presence of hydrophobic components at its cell surface, such as proteins also known as adhesins, which are important factors promoting strong adhesion to hydrophobic surfaces [58,65]. Furthermore, Maikranz et al. recently showed that *S. aureus* binds with many weakly binding macromolecules to hydrophobic surfaces, while only a few selected but strong macromolecules bind to hydrophilic surfaces. It is also suggested that the hydrophobic molecules form rather fast bonds while the hydrophilic binding molecules have to overcome a potential barrier [66], which may explain the decreased adhesion of cells on hydrophilic surfaces such as the PTU/ZnO composites produced in this study. Another reason that could explain the differences between PDMS and the PTU/ZnO composites is the surface charge of the material. The cell surface of *S. aureus* is moderately negatively charged (−4 to 6 mV) [67,68], thus, slightly negatively charged surfaces could be colonized by *S. aureus* cells, as long as van der Waals forces or other forces overcome the repulsion [69]. Furthermore, a study by Gross et al. suggests that increasing the repulsive forces between *S. aureus* and the polymer surface might complicate biofilm formation [70]. Thus, the fact that PTU/ZnO composites have a more negatively charged surface is also an important factor that can notably explain the smaller percentages of adhesion forces on these surfaces compared to PDMS. Another factor that seems to influence cell adhesion in the case of *S. aureus* is the stiffness of the materials. Only a few studies have reported on the influence of material stiffness on cell adhesion, but no consistent trend has been found on the subject [71]. For example, Lichter et al. found that *S. epidermidis* adhered better to stiff polyelectrolyte multilayered thin films [72], while Wang et al. found that the adhesion of *S. aureus* was reduced on polyacrylamide hydrogels with increasing stiffness [73]. In our case, we show that adhesion to stiffer materials is reduced, as the adhesion force recorded on PDMS is much higher than the one recorded on PTU variations. The stiffnesses of the PTU variations are not significantly different from each other except in the case of PTU containing 5 wt.% of t-ZnO, which is stiffer than the other PTU/ZnO composites and where the adhesion force recorded is significantly lower (86 ± 120 pN). While it is difficult to evaluate the independent effects of each parameter (charge, wetting property and stiffness) on the cell adhesion, in this case, we could suggest the hypothesis that at similar charge and WCA, an increased stiffness of the material might reduce the adhesion of cells onto it.

**Figure 2:**
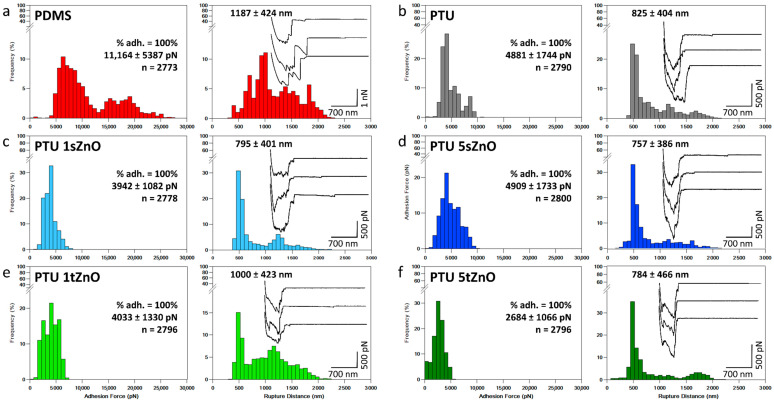
Single-cell force spectroscopy to probe the interactions between *C. glabrata* and material surfaces. Adhesion force histogram and corresponding rupture distance histogram obtained for seven cells interacting with (**a**) PDMS surface, (**b**) PTU surface, (**c**) PTU containing 1 wt.% of s-ZnO, (**d**) PTU containing 5 wt.% of s-ZnO, (**e**) PTU containing 1 wt.% of t-ZnO and (**f**) PTU containing 5 wt.% of s-ZnO. Insets in rupture distance histograms show representative force curves obtained in each case. % adh. is the % of force curves presenting adhesive events. Data were recorded using a set-point of 0.25 nN.

The same type of experiment was conducted with *C. glabrata*. Given the large size of these cells compared to *S. aureus* (five times larger), they were directly attached to tipless cantilevers and used to probe the interactions with the different material variations. The adhesion force and rupture histograms obtained are presented in Figure 2. As for *S. aureus*, in this case PDMS also shows the highest adhesion force of 11,164 ± 5387 pN and the highest rupture distance of 1187 ± 424 nm among the material variations (Figure 2a), including PDMS and PTU/ZnO composites. In comparison, pure PTU shows a significantly lower average adhesion force with 4881 ± 1744 pN and a corresponding rupture distance of 825 ± 404 nm (Figure 2b). With addition of 1 wt.% of t-ZnO and s-ZnO, the average adhesion force and rupture distance values only change slightly, whereas the addition of 5 wt.% of s-ZnO raises the adhesion force to 4909 ± 1733 pN. These results are summarized in Table 6. Statistical tests showed that the adhesion forces are significantly different from each other (*p*-value < 0.01), with an exception made by PTU with a filler content of 5 wt.% of s-ZnO, which is not significantly different from pure PTU. Thus, the results that are obtained in the case of *C. glabrata* show the same tendency as the ones obtained with *S. aureus*: the adhesion of the cells is strongly decreased on all PTU variations compared to PDMS. However, for *C. glabrata*, increasing the amount of spherical ZnO particles present in PTU does not seem to further decrease cell adhesion, but it does in the case of PTU filled with 5 wt.% of t-ZnO, where the lowest adhesion force is recorded. This was already the case for *S. aureus*. Moreover, it is interesting to note that compared to *S. aureus*, the amount of force curves presenting adhesive events for all investigated polymer surfaces was 100%, and that both adhesion forces and rupture distance values were higher for *C. glabrata*. This is probably due to the increased size of cells (approximately 5 µm in diameter), which, thus, increases the surface contact between the cells and the material, thus explaining the higher forces recorded. In this case, the long rupture distances measured and the presence of multiple peaks located after the contact point on the retract force curves also show that specific bonds are at play in the interaction. Here, strengthening the initial physico-chemical bonds also resulted in a transition from reversible to irreversible attachment [5].

The reasons for these lower adhesion values on PTU and PTU/ZnO variations compared to PDMS are probably the same as discussed previously in the case of *S. aureus*, i.e., lower water contact angle, higher negatively charged surface and higher stiffness of the PTU/ZnO composites. In particular, in the case of yeast cells, a study by El-Kirat-Chatel et al. demonstrated that the hydrophobic surface chemistry of the substrates plays an important role in the adhesion of the *C. glabrata* [60]. It shows that the adhesion of *C. glabrata* is mostly promoted by adhesins binding to hydrophobic surfaces. Which means that *C. glabrata* adheres more strongly to hydrophobic surfaces. This is in line with our findings. Regarding the surface charge of the materials, *C. glabrata* was found to bear a surface **ζ**-potential of −24 mV [74]. Thus, in this case, the repulsion of *C. glabrata* may also be more pronounced on PTU/ZnO composite surfaces than on PDMS, which has a smaller negative surface charge. Thus, perhaps explaining in part the reduced adhesion forces, although in this case, the percentage of force curves presenting adhesive events is still 100% for PTU/ZnO variations. Finally, regarding the stiffness of the materials, as far as we are aware, no studies have reported its influence on yeast cell adhesion. For PTU/ZnO composites, the adhesion force recorded is the lowest in the case of PTU with a filler content of 5 wt.% of t-ZnO particles. While the surface charge and the water contact angle are similar for all PTU/ZnO composites, the stiffness is significantly different for PTU/ZnO with 5 wt.% of t-ZnO. Thus, also in this case, it seems that stiffness is an important parameter to control the adhesion of microbes. 

## 4. Conclusions

**Figure 3:**
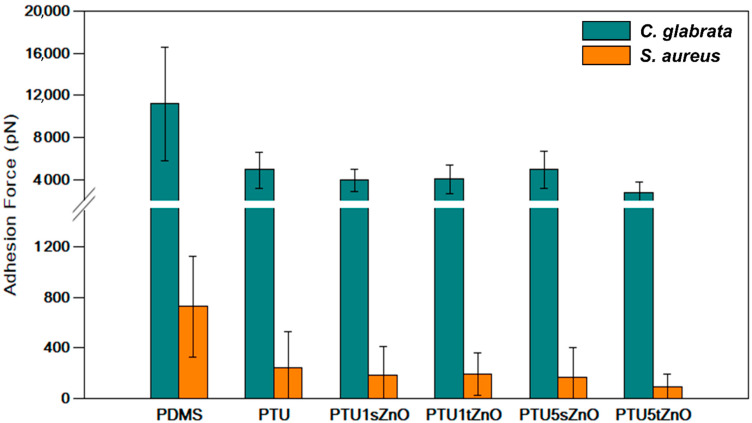
Recapitulative histogram showing the compared adhesion forces of *S. aureus* (orange bars) and *C. glabrata* (blue bars) to PDMS, PTU and PTU/ZnO composites.

Here, we demonstrate the development and characterization of new anti-fouling materials based on PTU filled with ZnO particles. These materials demonstrate promising anti-adhesion properties. Using single-cell force spectroscopy, we were able to show that the adhesion of two medically-relevant microorganisms, *S. aureus* and *C. glabrata* towards PTU/ZnO variations is successfully reduced when compared to PDMS (Figure 3). PTU/ZnO based materials exhibit a hydrophilic surface and a negative electro-static potential compared to conventional PDMS, which impedes the adhesion of *S. aureus* and *C. glabrata,* explaining the low adhesion forces compared to PDMS. Furthermore, the adhesion force recorded was significantly reduced for both microorganisms in the case of PTU containing 5 wt.% of t-ZnO, which might be the result of the increased stiffness of this particular variation compared to the others. Thus, this study shows that PTU/ZnO composite materials are efficient at reducing the initial attachment of microbial cells, and therefore show great potential for biomedical applications.

## Figures and Tables

**Table 1:** Surface roughness (*R_a_*) of polymer variations containing 0 wt.%, 1 wt.% and 5 wt.% of t-ZnO and s-ZnO particles measured by confocal laser-scanning microscopy (CLSM) and atomic force microscopy (AFM).

	*R_a_* Measured by CLSM (nm)		*R_a_* Measured by AFM (nm)	
**PDMS**	110 ± 10		13.60 ± 4.60	
**PTU**	110 ± 40		0.40 ± 0.08	
**Type of particle**	s-ZnO	t-ZnO	s-ZnO	t-ZnO
**PTU/1 wt.%**	100 ± 10	150 ± 10	0.15 ± 0.34	0.41 ± 0.07
**PTU/ 5 wt.%**	140 ± 30	210 ± 50	0.78 ± 0.20	0.72 ± 0.18

**Table 2:** Elastic modulus from tensile tests for PTU and PTU/ZnO variations.

Material Variation	Elastic Modulus (MPa)
PDMS	120 ± 9
PTU	624 ± 63
PTU 1 wt.% s-ZnO	641 ± 31
PTU 5 wt.% s-ZnO	630 ± 24
PTU 1 wt.% t-ZnO	611 ± 56
PTU 5 wt.% t-ZnO	689 ± 32

**Table 3:** Water contact angle measurements of polymer variations.

	PTU	PTU/1 wt.% t-ZnO	PTU/5 wt.% t-ZnO	PTU/1 wt.% s-ZnO	PTU/5 wt.% s-ZnO	PDMS
Water Contact Angle (°)	78.6 ± 2.4	77.9 ± 1.3	75.1 ± 1.7	76.0 ± 1.6	73.3 ± 2.3	114.9 ± 1.4

**Table 4:** **ζ**-Potential after 1 h immersion in KNO_3_ 10^−3^ M.

Material Variation	ζ Zeta Potential (mV)
PDMS	−3.5 ± 0.6
PTU	−23.0 ± 3.0
PTU/5 wt.% t-ZnO	−23.5 ± 0.9
PTU/5 wt.% s-ZnO	−22.0 ± 1.0

**Table 5:** Mean adhesion forces and rupture distances of *S. aureus* on a variation of six different material surfaces. The right column presents the percentage of curves presenting adhesive events.

Material Variation	Adhesion Force (pN)	Rupture Distance (nm)	% of Adhesion
PDMS	726 ± 397	212 ± 116	100%
PTU	237 ± 289	191 ± 146	89.5%
PTU/1 wt.% s-ZnO	177 ± 237	122 ± 77	88.5%
PTU/1 wt.% t-ZnO	191 ± 256	169 ± 113	87.2%
PTU/5 wt.% s-ZnO	165 ± 240	98 ± 72	77.7%
PTU/5 wt.% t-ZnO	86 ± 120	103 ± 61	40.4%

**Table 6:** The mean value of the adhesion force and the rupture distance of *C. glabrata* on a variation of six different material surfaces. The right column presents the percentage of curves presenting adhesive events.

Material Variation	Adhesion Force (pN)	Rupture Distance (nm)	% of Adhesion
PDMS	11,164 ± 5387	1187 ± 424	100%
PTU	4881 ± 1744	825 ± 404	100%
PTU/1 wt.% s-ZnO	3942 ± 1082	795 ± 401	100%
PTU/1 wt.% t-ZnO	4033 ± 1330	1000 ± 423	100%
PTU/5 wt.% s-ZnO	4909 ± 1733	757 ± 386	100%
PTU/5 wt.% t-ZnO	2684 ± 1066	784 ± 466	100%

## Data Availability

The data presented in this study are available on request from the corresponding author.

## Supplementary Materials

The following are available online at https://www.mdpi.com/2079-4991/11/2/271/s1, Figure S1: SEM micrographs of the different PTU/ZnO composites used in this study. Scale-bar = 100 µm, Figure S2: AFM height images of PDMS, PTU and PTU/ZnO composites. Scale-bar = 5 µm, Figure S3: Water contact angles photographs of PDMS, PTU and PTU/ZnO composites. Scale-bar = 500 µm, Table S1: Zn/C ratio on material surface for variations of ZnO particles in the PTU matrix measured by EDX, Table S2: Zn^2+^ concentration on material surface for variations of ZnO particles in the PTU matrix measured by Zinc ion release method with zincon.
