# Supplementary material for: Development of Polythiourethane/ZnO-Based Anti-Fouling Materials and Evaluation of the Adhesion of Staphylococcus aureus and Candida glabrata Using Single-Cell Force Spectroscopy
Source: Nanomaterials (Basel). 2021 Jan 21;11(2):271. doi: 10.3390/nano11020271 (PMC7909824; doi:10.3390/nano11020271)
Supplement: Supplementary file 1 [file nanomaterials-11-00271-s001.pdf]

## Supplementary materials

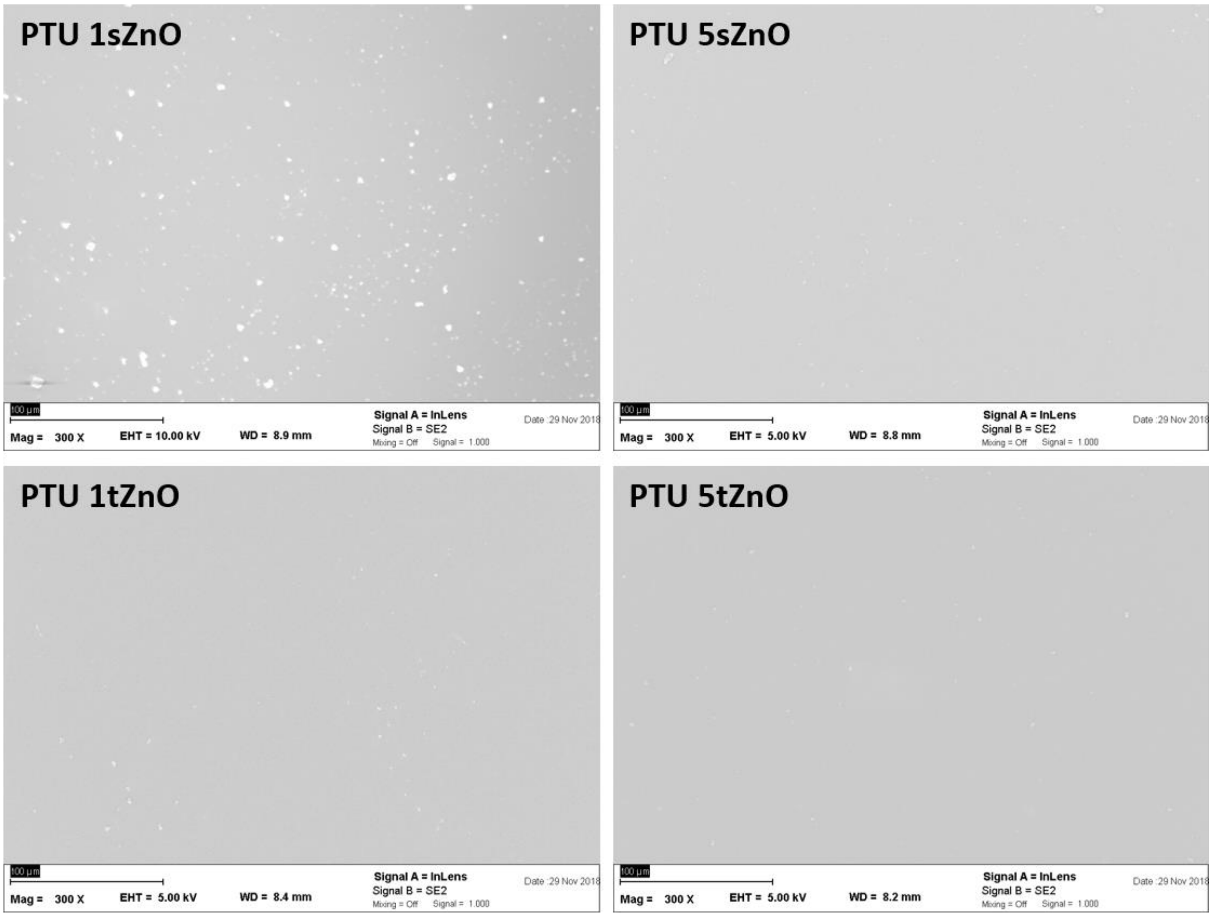


**Figure S1.** SEM micrographs of the different PTU/ZnO composites used in this study. Scale-bar = 100 µm.

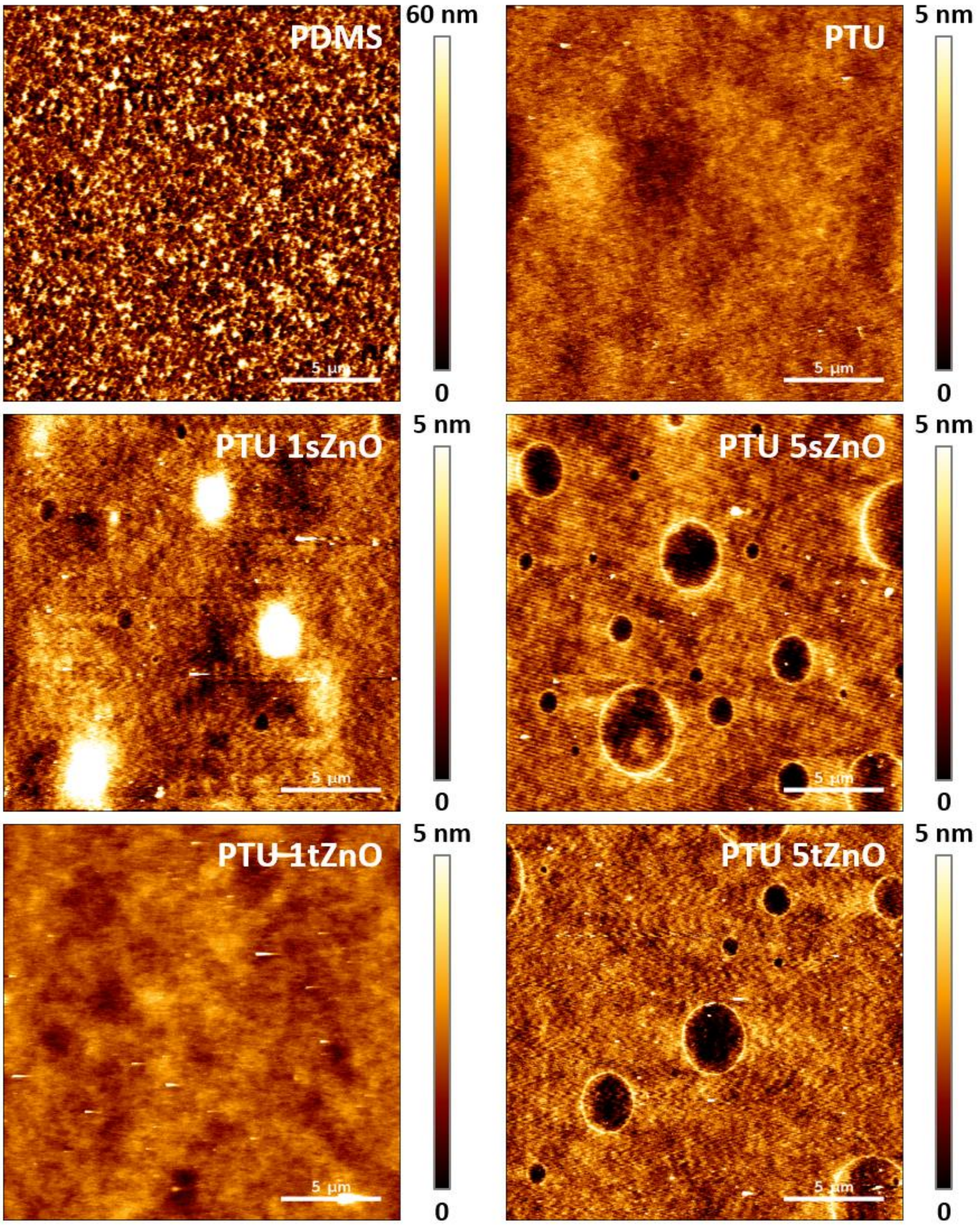


**Figure S2.** AFM height images of PDMS, PTU and PTU/ZnO composites. Scale-bar = 5 μm

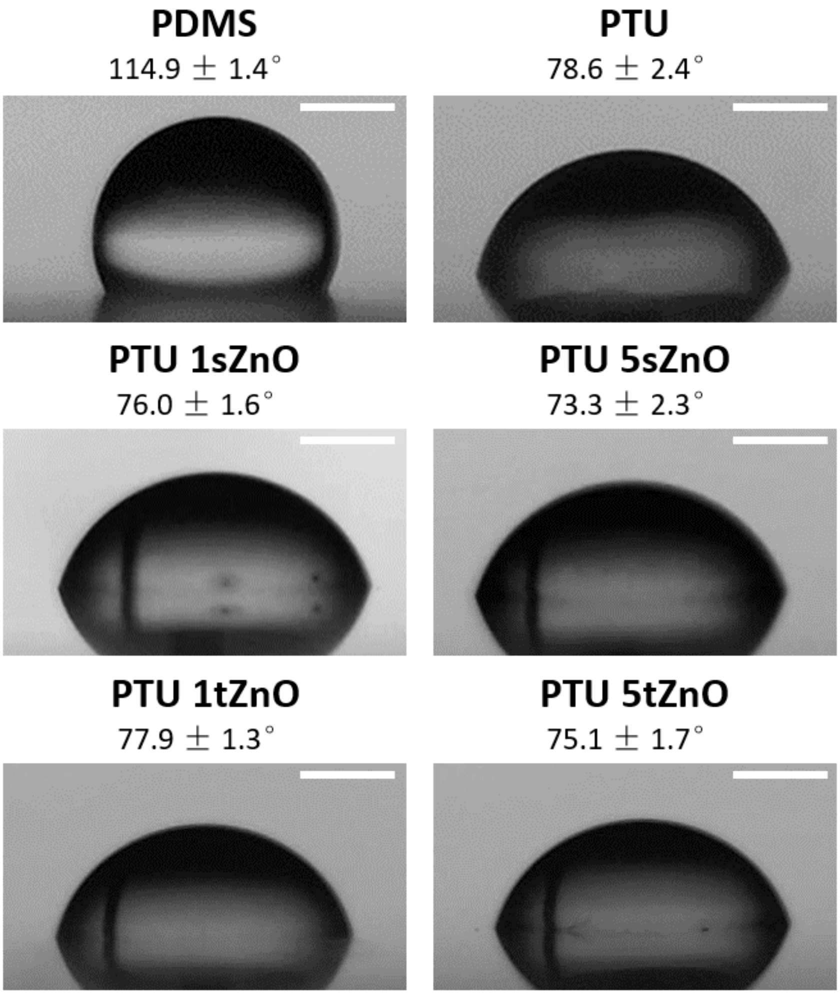


**Figure S3.** Water contact angles photographs of PDMS, PTU and PTU/ZnO composites. Scale-bar = 500 μm.

**Table S1.** Zn/C ratio on material surface for variations of ZnO particles in the PTU matrix measured by EDX. As Zinc (ZnO) is only contained in ZnO and Carbon (C) is only contained in PTU, the amount of ZnO present on the surface was determined by the Zn-to-C (Zn/C) ratio.

| Material Variation | Zn/C |
|---|---|
| PTU/1 wt.% t-ZnO | ND |
| PTU/1 wt.% s-ZnO | 0,008 |
| PTU/5 wt.% t-ZnO | ND |
| PTU/5 wt.% s-ZnO | ND |

**Table S2.** $Zn^{2+}$concentration on material surface for variations of ZnO particles in the PTU matrix measured by Zinc ion release method with zincon.

| Material Variation | $Zn^{2+}$ (mg/mL) |
|---|---|
| PTU/1 wt.% t-ZnO | ND |
| PTU/1 wt.% s-ZnO | ND |
| PTU/5 wt.% t-ZnO | ND |
| PTU/5 wt.% s-ZnO | ND |
